# Dosimetric and Clinical Risk Factors for the Development of Maxillary Osteoradionecrosis in Adenoid Cystic Carcinoma (ACC) Patients Treated With Carbon Ion Radiotherapy

**DOI:** 10.3389/fonc.2022.829502

**Published:** 2022-03-02

**Authors:** Barbara Vischioni, Stefania Russo, Martino Meuli, Maria Bonora, Sara Ronchi, Rossana Ingargiola, Anna Maria Camarda, Sara Imparato, Lorenzo Preda, Mario Ciocca, Silvia Molinelli, Ester Orlandi

**Affiliations:** ^1^Radiation Oncology Clinical Department, National Center for Oncological Hadrontherapy, Pavia, Italy; ^2^Department of Clinical, Surgical, Diagnostic and Pediatric Sciences, University of Pavia, Pavia, Italy; ^3^Division of Radiotherapy, Istituto Europeo di Oncologia (IEO) European Institute of Oncology, Istituto di Ricovero e Cura a Carattere Scientifico (IRCCS), Milan, Italy; ^4^Department of Oncology and Hemato-Oncology, University of Milan, Milan, Italy; ^5^Radiology Institute, Fondazione Istituto di Ricovero e Cura a Carattere Scientifico (IRCCS) Policlinico San Matteo, Pavia, Italy

**Keywords:** ACC, maxillary osteonecrosis, carbon ion radiotherapy, risk factors, late toxicity

## Abstract

**Background:**

The present study aims to evaluate dosimetric and clinical risk factors for the development of maxillary osteoradionecrosis (ORN) in head and neck adenoid cystic carcinoma (ACC) patients treated with carbon ion radiotherapy (CIRT).

**Methods:**

Clinical data and treatment plans of ACC patients, consecutively treated from January 2013 to September 2016 within the phase II clinical trial CNAO S9/2012/C, were retrospectively reviewed. ORN and other treatment-related toxicity were graded according to the Common Terminology Criteria for Adverse Events (CTACE), version 4.0. The maxillary bone was contoured on the planning CT, and only patients receiving more than 10% of the prescription dose at their maxilla were considered for the analysis (67 patients). The volumes of maxilla receiving doses from 10 Gy (RBE) to 60 Gy (RBE) (V_D_), with an increment of 10 Gy (RBE), and additional clinical factors were correlated to the incidence of ORN with univariate analysis (Chi-square test). The logistic regression model was subsequently applied for multivariate analysis. Treatment plans calculated with a local effect model (LEM)-based optimization were recalculated with the modified microdosimetric kinetic model (MKM), and compared with literature data from the Japanese experience.

**Results:**

The median time interval from the start of CIRT to ORN appearance was 24 months (range, 8–54 months). Maxillary ORN was observed in 11 patients (16.4%). Grade 1 ORN was observed in 2 patients (18.1%), G2 in 4 (36.3%), G3 in 4 (36.3%) and G4 in 1 (9.3%). From univariate analysis, the site of the tumor, the presence of teeth within the PTV and acute mucositis correlated with the development of maxillary ORN. V_D_ were significantly higher for all the dose levels tested in patients with maxillary ORN than patients without necrosis, according to both radiobiological models. The multivariate analysis showed that V60 significantly correlated with ORN risk.

**Conclusion:**

The volume of maxilla irradiated with high dose values was relevant for ORN development in our cohort of ACC patients. These results are in line with previously published data obtained with a different radiobiological model. Our findings might be helpful to prevent the risk of ORN in patients receiving CIRT.

## Introduction

Osteoradionecrosis (ORN) is described as a chronic wound that fails to heal with bone exposure. It is a treatment related complication primarily reported in the head and neck population treated with radiotherapy (RT) frequently associated with a substantial morbidity causing pain and infection. The pathophysiology of its occurrence is linked to the hypoxia, hypocellularity, and hypo vascularity that follow a RT course (1). There are several risk factors that have been reported to favor ORN in patients undergoing RT, for example alcohol and tobacco consumption, dental and periodontal status deterioration, trauma after dental extractions, oral implantology, insufficient body mass index, general comorbidities such as diabetes, and anti-inflammatory or anti-coagulant therapy (2). Treatment related factors that might be linked to ORN are technique of irradiation, dosimetric parameters, fractionation schedule, extension of irradiated volumes, and concomitant chemotherapy (3).

The incidence of ORN of the mandible when irradiating parotid tumors and also at the skull base when irradiating tumors localized at the nasopharynx, has decreased in recent years with the transition from 2 and 3D-RT to the modern intensity modulated-RT (IMRT), since the more localized deposition of the higher doses to the tumor target in respect to the surrounding normal tissues (4, 5). In this regard, carbon ion RT (CIRT) has shown its superiority in dosimetric studies in delivering tumoricidal dose while sparing the surrounding normal tissues, due to the sharp penumbra of the therapeutic beams. Furthermore, the radiobiological properties of CIRT that causes not repairable DNA damage not cell cycle specific nor oxygen dependent (6), have advocated its therapeutic use for radioresistant tumors, such as adenoid cystic carcinoma (ACC). The gold standard treatment for ACC is radical surgical resection followed by post-operative RT (7). In case of unresectable locally advanced tumors or with tumor macroscopic persistence after surgical resection or patient contraindication to surgery (8–10), or reirradiation (11), CIRT has been recently shown to have higher efficacy compared to conventional photon RT (12).

While numerous clinical and physical factors have been reported to be associated with ORN development after photon RT, the risk factors for maxillary ORN after CIRT are still under investigation. Sasahara et al. (13) reported V50 as a good predictor of ORN in multivariate analysis in a series of 63 malignant radioresistant tumors (namely, 24 ACC) treated with CIRT at a prescribed dose of 57.6 Gy (RBE) in 16 fractions, with passive beam delivery. RBE-weighted dose calculation was based on the semi-empirical model by Kanai et al. (14), while the modified microdosimetric kinetic model (MKM) is currently used for pencil beam scanning in Japan (15). The two models have been validated for consistency, and only the latter will be adopted here for comparison against available literature data.

The aim of the present study was to identify dosimetric and non-dosimetric risk factors correlated to ORN development for head and neck ACC patients treated at CNAO (National Center for Oncological Hadrontherapy, Pavia, Italy) with CIRT delivered with curative intent. At CNAO the local effect model (LEM—version I) is employed for RBE calculation as in other European CIRT centers (16), with treatment protocols derived from the Japanese experience (17), after mice and cell experiments to assure comparable radiobiological efficacy of the CNAO carbon ion beam to the reference CIRT beam of the NIRS Hadrontherapy Center in Japan (18). The impact of RBE conversion from MKM to LEM on prescription doses and organ at risk (OAR) constraints was extensively reported for different CIRT cancer indications, including ACC (19). Only MKM model-based analysis on ORN has been reported so far for patients with head and neck tumors (13), while here we directly investigate risk factors for ORN development when irradiating head and neck patients with CIRT by using the LEM model. The findings from the present study might be helpful to prevent ORN risk at the maxilla in patients receiving CIRT in facilities where the LEM is used for treatment of patients in 16 fractions, directly estimating the dose that might be delivered without causing ORN, and thus avoiding the deterioration of quality of life reported for example when ORN evolves into oronasal fistula (20). Furthermore, in our study the identification of other clinical and treatment related risk factors predisposing to ORN might be helpful to assess ORN risk in the head and neck population undergoing CIRT and plan adequate follow up schedule with possible preventive measures.

## Material and Methods

### Study Protocol Design

The present study is a retrospective, observational study, a subanalysis of the prospective phase II clinical trial ongoing from January 2013 to September 2016 at CNAO (CNAO S9/2012/C) to assess safety and feasibility of CIRT with radical intent for ACC patients with partially resected tumors (R1/R2 margins), or unresectable tumors, or unfit to surgery (21). CNAO’s Ethics Committee formally approved the present research protocol CNAO OSS/19/2020, and the patient consents for the research participation were properly collected and stored. The primary endpoint of the present study was to evaluate the dose of CIRT associated with the development of ORN at the maxillary bone in the ACC patients treated at CNAO with CIRT according to the LEM model. Secondary endpoints were the correlation of ORN in our series with the patient clinical and treatment characteristics to describe possible factors that might predict bone toxicity after CIRT.

### Patient’s Baseline and Follow-Up Data

A total of 128 patients with head and neck ACC consecutively treated with CIRT along with their diagnostic imaging and treatment plan data were screened for selection in the present study. Only the patients with at least more than 10% of the total prescription dose of 68.8 Gy (RBE) to their maxilla were selected for the analysis as detailed in Sasahara et al. (13). On the other side, patients with tumor invading the maxillary bone before CIRT, or with maxillary recurrence after CIRT, were excluded to avoid confounding factors for ORN assessment. At the end, 67 patients, 35 women (52.2%) and 32 men (47.8%) were considered for the analysis. The mean age of the studied population at the beginning of the CIRT course was of 55 years. Patients clinical and tumor characteristics (namely, anamnestic data that have been reported to impact on ORN except for osteoporosis), and data related to previous surgery and CIRT toxicity, were collected and detailed in **Table 1**. Most of the tumors were in the sinonasal area (35.8%). The other sites involved were the parotid glands (20.9%), the pharynx (17.9%), the submandibular glands (13.4%), the oral cavity (9%), and the external auditory channel (3%). Tumors were all re-staged following the 8th edition of TNM staging system (AJCC/UICC). Prior to therapy, the patients were adequately informed about the possible risks of maxillary ORN as a side effect after irradiation. As of institutional practice, a complete dental examination and a dental–periodontal prophylaxis were requested. If indicated, atraumatic extraction of dental elements with a poor prognosis was performed at least 10 days prior to treatment. For each patient, a dental health certificate stating the good dental health was mandatory to start CIRT. No patient underwent bisphosphonates or concomitant systemic treatment during CIRT or adjuvant chemotherapy after CIRT. After CIRT patients were regularly followed up every 3 months for at least 2 years, then every 6 months since the fifth year, with magnetic resonance imaging (MRI) examination and fibroscopic local examination, with a staging total body Computed Tomography (CT) once per year. The diagnosis of maxillary ORN relied on the clinical symptoms reported by each patient, the oral-maxillo-facial physical examination, and the imaging scan acquired during the follow up reviewed by a senior radiologist. In case of maxillary ORN a maxillo-facial CT scan was also performed along with the follow up MRI to further study the affected bone. Acute and late toxicities were collected at each follow up clinical visit and scored according to the Common Terminological Criteria for Adverse Events (CTCAE), version 4 (22), including bone toxicity.

**Table 1:** Patients clinical and tumor characteristics.

Clinical factors	Subgroup	N (%)
Age	<57	33 (49.3)
	≥57	34 (50.7)
Sex	Male	32 (47.8)
	Female	35 (52.2)
Diabetes	Yes	6 (9.0)
	No	61 (91.0)
Autoimmune disease	Yes	2 (3.0)
	No	65 (97.0)
Hypertension	Yes	19 (28.4)
	No	48 (71.6)
**Tumor characteristics**	**Subgroup**	**N (%)**
Tumor Site	Sinonasal	24 (35.8)
	Others	43 (64.2)
T Stage	T1	5 (7.5)
	T2	15 (22.4)
	T3	6 (9.0)
	T4	41 (61.2)
N Stage	N0	62 (92.5)
	N1	2 (3.0)
	N2	3 (4.5)
M Stage	No	61 (91.0)
	Lungs	5 (7.5)
	Liver	1 (1.5)
Surgery before CIRT	Yes	43 (64.2)
	No	24 (35.8)
Margins status at surgical report	RX	8 (11.9)
	R1	31 (46.3)
	R2	4 (6.0)
	No Surgery	24 (35.8)
Residual tumor disease (Pre CIRT MRI)	Yes	30 (44.8)
	No	37 (55.2)
**Toxicity after CIRT**	**Subgroup**	**N (%)**
Maximum acute toxicity during Follow Up	G0	0 (0.0)
	G1	17 (25.4)
	G2	37 (55.2)
	G3	13 (19.4)
Acute mucositis	Yes (G1-G4)	49 (73.1)
	No (G0)	18 (26.9)
Maxillary ORN	Yes	11 (16.4)
	No	56 (83.6)

### Carbon Ion RT Treatment Planning and Dose-Volume Histogram Analysis

A set of 2 mm thickness CT scan was acquired for treatment planning. Patients were immobilized with a personalized thermoplastic head–neck–shoulder mask equipped with bite-blocks and fixation points to the couch as previously reported (23). After the simulation CT, a MRI was acquired in the same setup condition. The gross tumor volume (GTV) was first outlined based on the morphological analysis of the planning CT after registration with MRI, namely, the primary tumor lesion or the surgical tumor bed, and the metastatic lymph nodes. The high-risk clinical target volume 1 (CTV1) was delineated by including the GTV and a minimum of 5 mm margin in all directions to cover the microscopic extent of the lesion. A larger CTV2 (including the CTV1) with the anatomical area at risk of perineural spread along the cranial nerves up to the skull base was also outlined. Irradiation to prophylactic lymph nodes was omitted. PTV1 and PTV2 were created, adding a 2 mm margin at each CTV. The total prescription dose was 68.8 Gy (RBE) in 16 fractions over 4 weeks (4.3 Gy (RBE)/fraction, 4 fractions/week). CTV2 was irradiated with a dose of 38.7 Gy (RBE) and the high-risk CTV1 was irradiated up to the total prescribed dose. Patients were treated with 2 to 4 fields with active pencil beam scanning technique. Treatment plans were optimized with Syngo PT (Siemens AG Healthcare, Erlangen, Germany) treatment planning system (TPS), and the LEM I model was used for RBE-weighted dose calculation with an ideal α/β ratio of 2 Gy. For this study, all treatment plans were recalculated with a research version of Raystation TPS (V6.99, Raysearch, Stockholm, Sweden), which provides both LEM I and MKM for RBE-weighted dose calculation.

The radiological morphology of the maxillary bone was first studied and then manually contoured on planning CT for each patient. The delineation of maxillary volumes included the alveolar process, the palatine process and the roots of the upper teeth in their own alveoli as previously reported (13). Pterygoid processes, sinus cavities and the crowns of the upper teeth were excluded. The mean maxillary volume in the patient series was 26.2 ml (range: 11.1–42 ml). The absolute maxillary volumes receiving doses D ranging from 10 to 60 Gy (RBE) were extracted from the dose volume histograms (DVHs) of the patients, and were expressed as V_D_ doses (V10, V20, V30, V40, V50, and V60), for both LEM and MKM calculated plans. For each V_D_ parameter of the LEM-based plans, the median value was calculated over the entire patient cohort, and patients dichotomized accordingly, to test the correlation with the incidence of ORN. Furthermore, the patient cohort was divided into two groups according to the appearance of ORN, and the average DVHs were calculated for both groups and radiobiological models (LEM and MKM). In the work by Sasahara et al., the absolute maxillary volume receiving 50 Gy (RBE) (V50_MKM_) was found significant for the onset of ORN. We therefore extracted the V50_MKM_ for each patient, from the MKM-DVH, and calculated the corresponding LEM-based dose received by the same volume of maxilla in each delivered plan, to compare our treated population with literature dosimetric data (13). For each LEM-based patient treatment plan, the presence of teeth in the irradiated PTV was recorded for analysis.

### Statistical Analysis

The Chi-square test was performed to investigate both clinical and dosimetric variables as predictors of ORN risk. For statistically significant variables only, the strength of association was estimated by Cramer’s V. The Kaplan–Meier method was adopted to evaluate the cumulative incidence of late post-radiation bone effects with respect to the only statistically significant variables in the univariate analysis. The difference that was observed between the cumulative incidence curves was compared with the Log-rank test. P-values <0.05 were considered statistically significant. The logistic regression model was subsequently applied to assess how the baseline risk of developing ORN varied according to the presence/absence of several given variables, namely, the V_D_ in 10 Gy (RBE) increments (V10–V60) extracted from the LEM-based calculated plans, and statistically significant clinical factors. To increase the power of our logistic regression model, we then limited the variables in the analysis to the V_D_ in 10 Gy (RBE) increments and the ORN incidence. If the estimated odds ratio was >1, the variable was assumed to be a risk factor because it increased the baseline risk. SPSS ver.19 software (IBM SPSS, IBM Corporation, Somers, NY) was used for statistics.

## Results

### Maxillary ORN and Risk Factors

The median follow-up time was of 50 months (range 8–82 months). ORN was observed in 11 patients (16.4%) with a median time to ORN development of 24 months (range 8–54 months). Grade 1 ORN was observed in 2 patients (18.1%), G2 in 4 (36.3%), G3 in 4 (36.3%), and G4 ORN in 1 patient (9.3%). In this latter case the patient had a microbial infection at the sphenoid bone not properly managed, and the necrosis expanded up to the right carotid channel approximately 10 months after CIRT. To avoid fatal bleeding due to carotid blow out, the patient had to perform carotid artery coiling. At the last follow up, the patient was alive without evidence of disease, and his necrosis stable. In 6 patients ORN started at the bone close to the teeth alveoli, and in one case followed the loss of one dental element. Majority of the cases had stable ORN at the last follow up (81.8%). In 2 cases with G3 ORN a surgical sequestrotomy was performed, while in one case the defect was repaired with a temporal muscle flap. Furthermore, 3 patients in the series took advantage of hyperbaric chamber treatment to stabilize the progression of their G2 necrosis after intermittent cycles of antibiotics for recurrent infections of the necrotic area.

On univariate analysis, the tumor site, and acute mucositis ≥G1 developed during CIRT were found to be statistically associated with maxillary ORN (**Table 2**). Majority of the patients with ORN had a tumor located in the sinonasal area (p-value 0.035). Among patients treated with CIRT who did not experience acute mucositis, none had maxillary ORN. Among those with acute mucositis, however, 22.4% also had a late effect of maxillary toxicity (p-value 0.028). Log-rank tests for these factors confirmed that the difference between the Kaplan–Meier curves was statistically significant. In fact, tumors located in the sinonasal area had a higher probability of developing ORN over time (p-value 0.053), as for patients experiencing acute mucositis during CIRT compared to the others (p-value 0.040).

**Table 2:** Correlation of relevant patients clinical and tumor characteristics and ORN at univariate analysis.

Clinical Factors	Subgroup	ORN rate N (%)	p-value
Age	<57	8 (24.2)	0.089
	≥57	3 (8.8)	
Sex	Male	5 (15.6)	0.867
	Female	6 (17.1)	
Diabetes	Yes	1 (16.7)	0.986
	No	10 (16.4)	
Autoimmune Disease	Yes	0 (0)	0.525
	No	11 (16.9)	
Hypertension	Yes	2 (10.5)	0.413
	No	9 (18.8)	
**Tumor Characteristics**	**Subgroup**	**ORN rate (%)**	**p-value**
Tumor Site	Sinonasal	7 (29.2)	0.035
	Others	4 (9.3)	
T Stage	T1	0 (0)	0.303
	T2–T4	11 (17.7)	
Surgery Before CIRT	Yes	6 (14,0)	0.466
	No	5 (20.8)	
Residual Tumor Disease (Pre CIRT MRI)	Yes	5 (16.7)	0.588
	No	1 (7.7)	
**Toxicity After CIRT**	**Subgroup**	**ORN rate (%)**	**p-value**
Acute Mucositis	G1–G4	11 (22.4)	0.028
	G0	0 (0)	

### Dose-Volume Risk Factors and Comparison With Published Data

**Figure 1:**
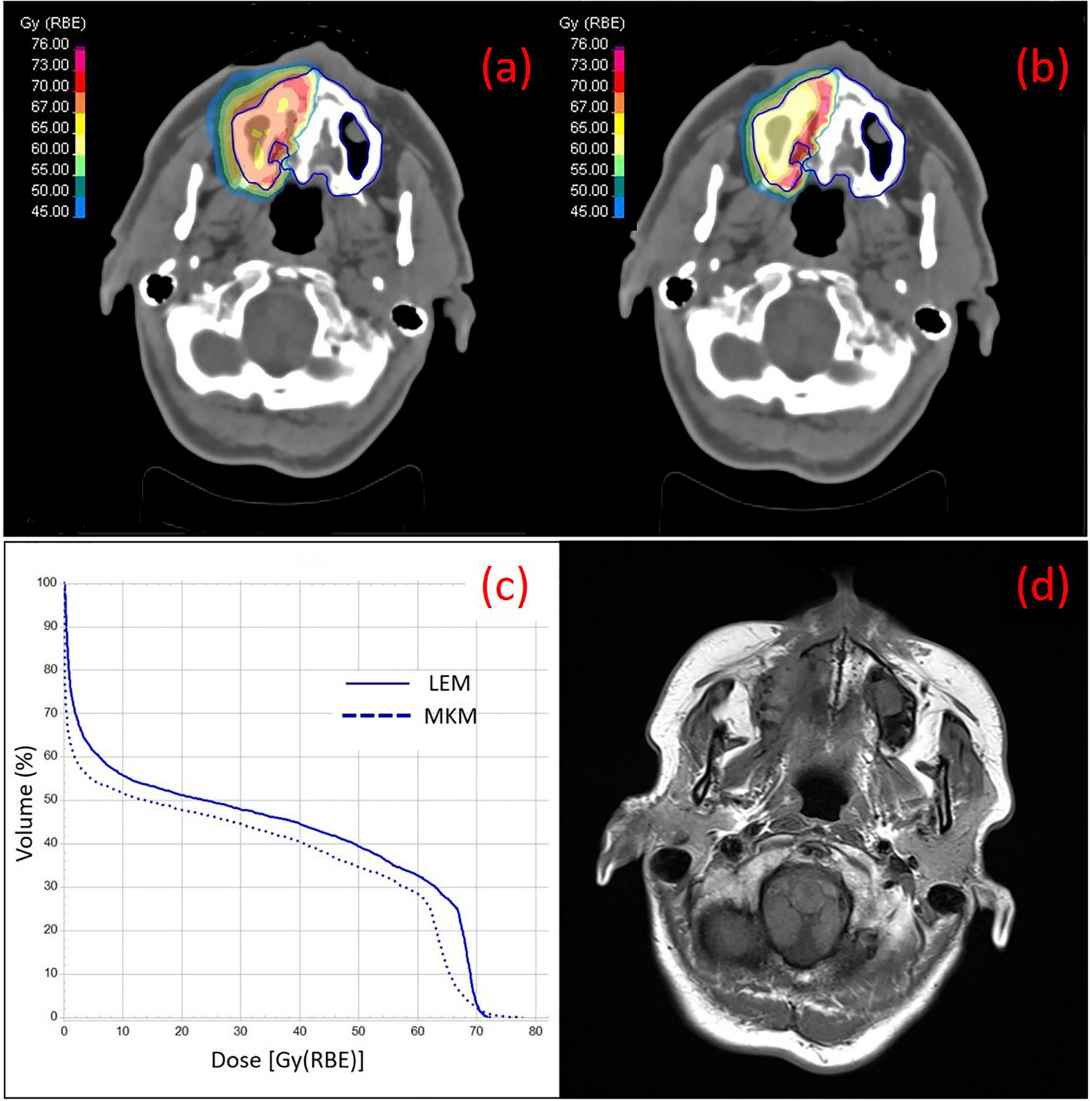
Treatment plans on the simulation CT of one of the patients of the series with ACC at the right nasal cavity with color wash of the most representative isodoses at the maxilla (contoured in blue), calculated with LEM **(A)** and MKM **(B)** radiobiological model, are shown. The irradiation geometry consisted of 2 beam ports with couch rotation of 165°C and 180°C. In **(C)** the DVH curves for the maxilla calculated with LEM and MKM are depicted. In **(D)** a T1-weighted axial image taken from the 2 years post-CIRT MRI of the same patient is depicted with typical imaging features of maxillary Grade 1 necrosis.

In **Table 3** are reported the median cut-off value for V10–V60 and the V_D_ correlation with the ORN incidence in our series as extracted from the LEM-based patients treatment plans. On univariate analysis, all the V_D_ were statistically associated with maxillary ORN (V10, V20, V30, V40, V50, V55, and V60) together with the presence of teeth in the irradiated PTV. The incidence of ORN always occurred in patients where the maxillary volume received dose higher than or equal to the median cut-off. **Figure 1** shows an example of one of the patients in the series that experienced ORN along with the screenshot from its treatment plans (**Figure 1**). Log-rank tests for all the V_D_ confirmed that the survival function from ORN decreases as the irradiated maxillary volume increases, both for low and high dose levels (data not shown). Among those patients who had no upper teeth within the PTV, none reported ORN. Among those who had maxillary teeth within the PTV, 36.7% suffered from maxillary ORN after CIRT (p-value <0.001).

**Table 3:** Univariate analysis of dichotomized dosimetric factors from LEM-based calculated treatment plans to assess dose-volume relationship and risk of maxillary ORN.

V-DOSE	Subgroup	ORN (%)	p-value
**V10**	<8.6	0	<0.001
	≥8.6	32.4	
**V20**	<5.5	0	<0.001
	≥5.5	32.4	
**V30**	<4.4	0	<0.001
	≥4.4	32.4	
**V40**	<2.9	0	<0.001
	≥2.9	32.4	
**V50**	<1.0	0	0.001
	≥1.0	31.4	
**V60**	<0.6	0	0.001
	≥0.6	31.4	
**Teeth in PTV**	Yes	36.7	<0.001
	No	0	

Teeth within the PTV were considered a risk factor for analysis.

Considering all the V_D_, the tumor site, the presence of teeth within the PTV and acute mucositis, the logistic regression model identified only V60_LEM_ as a significant variable in differential risk compared with baseline risk (Hazard Ratio = 1.431, p = <0.001, 95% CI 1,194–1,716). The risk of developing ORN increased by 43.1% if a maxillary volume ≥0.6 ml was covered with at least 60 Gy (RBE) dose. When limiting the variables in the regression analysis to the V_D_ in 10 Gy (RBE) increments to increase the power of prediction, the V60 _LEM_ was still identified as independent factor significantly associated to the ORN development in our series.

**Figure 2:**
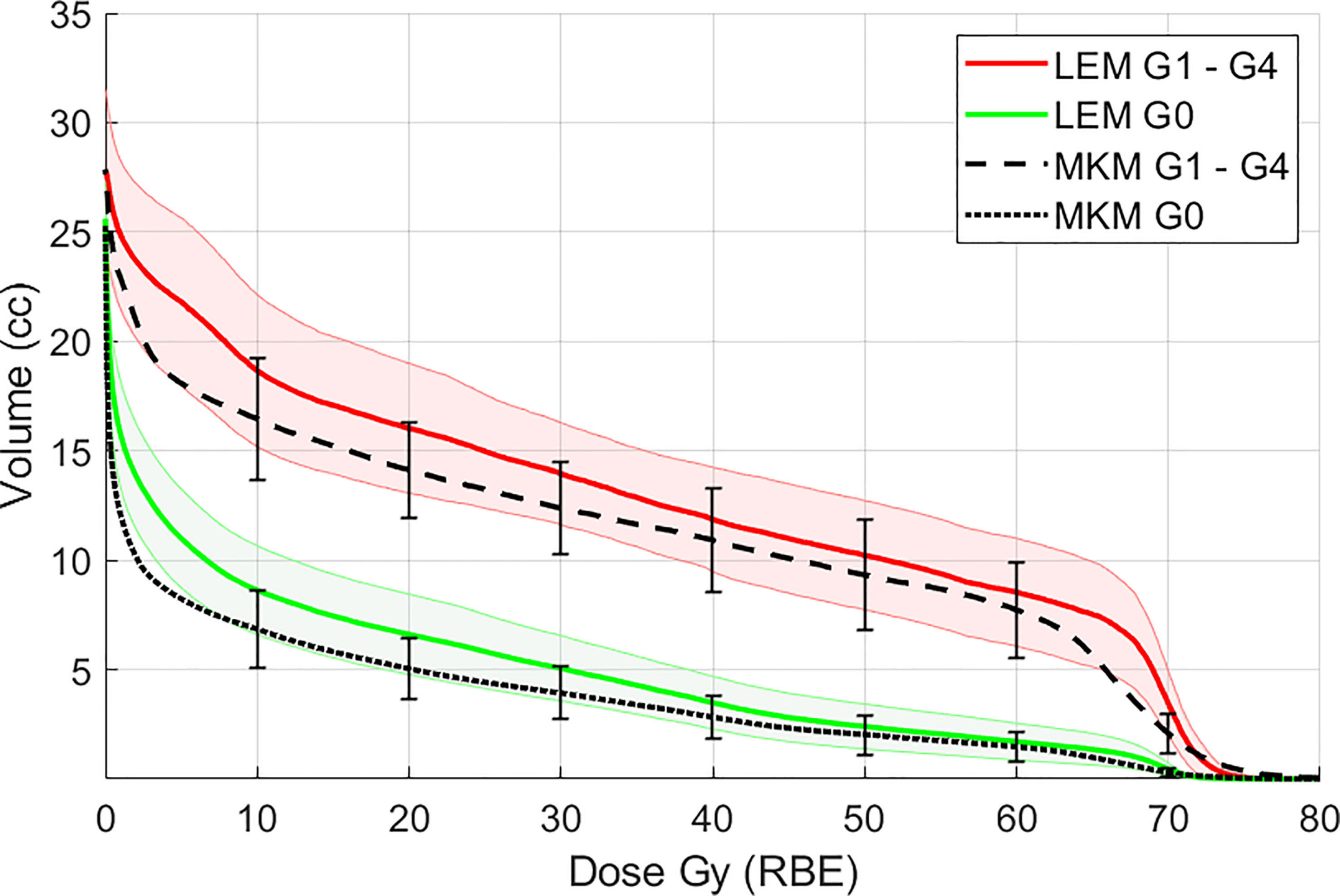
Mean maxillary volumes at incremental dose levels from 10 to 60 Gy (RBE) calculated with the LEM model in patients with ORN (G1–G4, red solid line) vs patients without ORN (G0, green solid line). In black the maxilla DVH averaged for all the patients for the MKM model is also presented. Shaded bands and vertical error bars represent 2 SEM (Standard Error of the Mean) for the LEM and MKM model, respectively.

**Figure 3:**
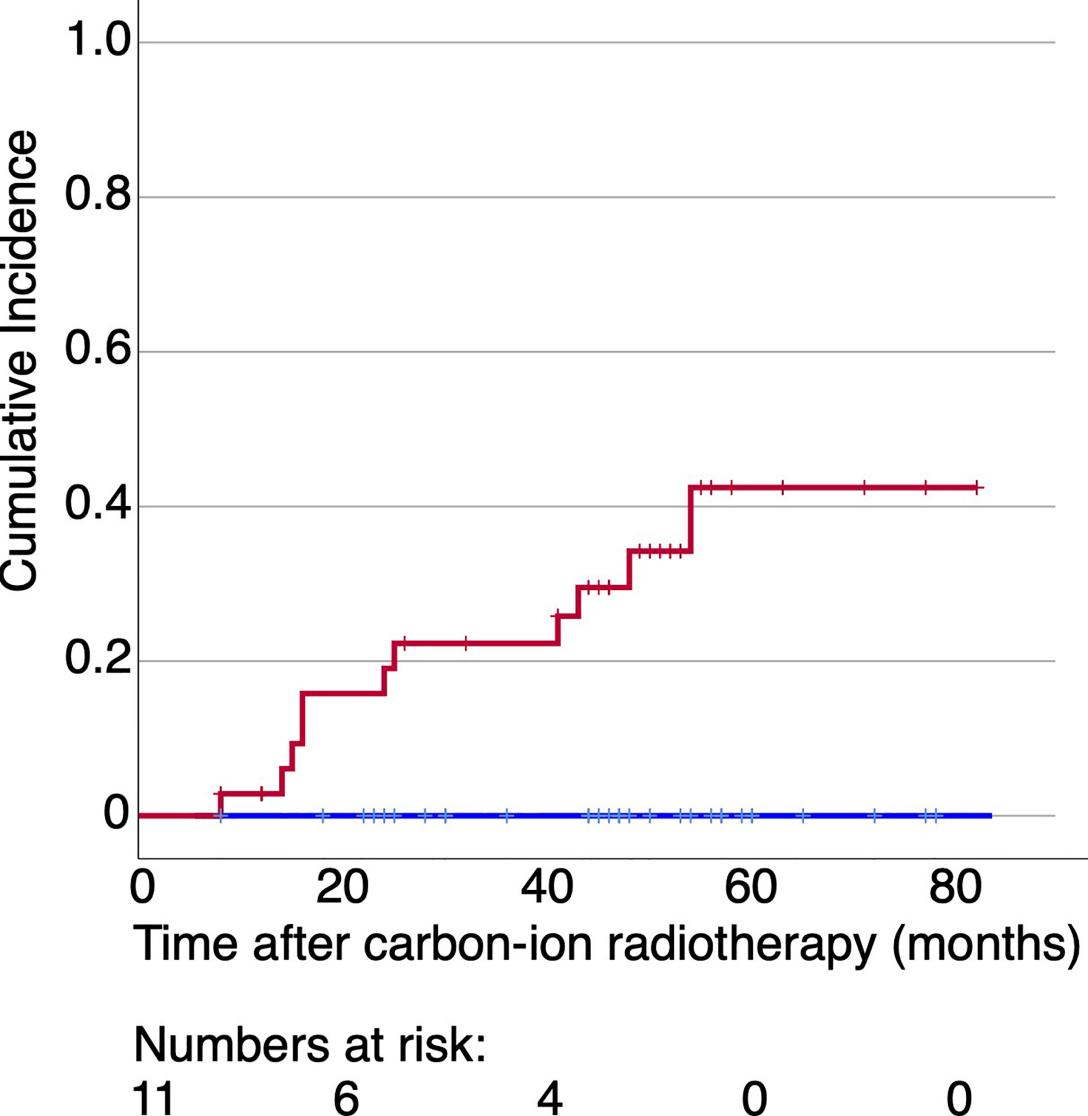
Cumulative incidence of CIRT-related ORN over time for the patients irradiated with more than 60 Gy (RBE) at 0.6 ml of their maxilla (red line) or not (blue line) with data extracted form LEM-based treatment plans. No patients experienced ORN in case the dose to 0.6 ml of their maxilla was below 60 Gy (RBE).

When the cohort of the patients was divided into two groups according to the appearance of ORN, the average maxillary dose received by patients with ORN was higher at all volume levels, in both RBE languages (**Figure 2** for both LEM and MKM treatment plans). In **Figure 3** we have shown the cumulative incidence of CIRT-related ORN over time in our series when ranking the patients according to the V60 _LEM_.

Finally, as for comparison with previous literature dosimetric data acquired with the MKM radiobiological model (13), the LEM-based dose received by the V50_MKM_ of each patient in our cohort averaged over the patient population, was 55 Gy (RBE) ± 2 SD.

## Discussion

Bone necrosis is an important side effect, not only when irradiating with conventional photon RT, but also after CIRT. Here we analyzed for the first time the risk factors for ORN development in patients treated with LEM-based CIRT. Although we had a small sample of patients and of ORN events, we have still shown in our series the relevance of dosimetric parameters in predicting ORN risk after CIRT, with both the biological models mostly used in CIRT facilities worldwide for treatment planning, the LEM and MKM model. Tumor treatment, especially when curative, must not disregard ORN risk to preserve patient quality of life. However, sparing dose to the maxilla, should never be prioritized at the cost of target coverage.

With a similar study design, in Sasahara et al. the V50_MKM_ was found to be a risk factor at multivariate analysis, along with the presence of teeth in the PTV, in a series with different radioresistant tumors including ACC treated with CIRT at a prescription dose of 57.6 Gy (RBE) (13). As pointed out in Musha et al., the adjustment for the RBE model is necessary in CIRT facilities outside Japan (24), when investigating for dosimetric factors predisposing to toxicity such as ORN. As part of our investigation, here we wanted to translate the V50_MKM_, significant factor for ORN reported for the Japanese series (13), into the corresponding V_D_ extrapolated from our LEM optimized plans recalculated with the MKM Japanese radiobiological model. In our patient population, V50_MKM_ corresponded to V55_LEM_. The statistical significance of 55 Gy (RBE) was not explicitly tested, but it is in good agreement with the dose value resulting significant at our multivariate analysis (V60_LEM_), considering all the uncertainties involved. As a limit, several sources of dose deviation need to be considered when comparing clinical outcomes from patient series treated with different techniques, such as the beam delivery, the different beam number per fraction and beam arrangements, physical and RBE-weighted dose calculation uncertainties, the small number of patients, and different clinical characteristics (19). Furthermore, the power of the multivariate analysis presented here is low because of the small number of ORN events. Indeed, we might consider this study as a pilot trial, and for the future we aim at increasing the number of patients to produce more solid data on the clinical and dosimetric factors predisposing to ORN.

The relevance of the high doses when irradiating with CIRT in comparison to photon RT was pointed out in Ikawa et al., where 76 patients with oral nonsquamous cell carcinoma treated with CIRT at mostly with 64 Gy (RBE) prescription dose in 16 fractions equivalent to 89.6 Gy (RBE) at a fractionation of 2 Gy (RBE) per fraction, experienced 11.8% G3 ORN requiring surgery (25). Furthermore, in Naganawa et al. reporting on CIRT in 19 patients with oral malignant mucosal melanoma treated with 57.6 Gy (RBE) prescription dose in 16 fractions, all Grade 2 and 3 ORN were derived from the alveolar bone in the high dose–irradiated volume. Despite this, the mean V50_MKM_ of the maxilla or mandible was 12.5 ml (range, 0–23.4 ml) for patients with Grade 0–1, and 12.5 ml (range, 3.1–22.3 ml) for patients with Grade 2–3 ORN, respectively, with a difference not statistically significant (26).

The impact of lower doses after CIRT on the mandible ORN onset has been recently reported to be significant when irradiating tumors localized at the oral cavity and oropharynx, for the V30_MKM_–V45_MKM_ at the mandible, and in case of V10_MKM_–V30_MKM_ to the teeth (24). In our study, all the V_D_ (V10 _LEM_–V60 _LEM_) tested at univariate analysis were statistically correlated with the occurrence of ORN. Maxillary ORN cases occurred in the subgroup of patients with V_D_ larger than- or equal- to the median volume for that DVH as it has been reported also in Sasahara et al. although with a different biological model (13). This indicates that the risk of ORN in our analysis of LEM-based calculated treatment plans might be enhanced both for larger bone volumes exposed to a low dose bath (<50 Gy (RBE)), and for smaller bone volumes exposed to a high dose >55 Gy (RBE). This finding might be translated into the importance of maximizing the dose conformity at the tumor target while sparing sensitive organs for toxicity, such as the maxilla, minimizing the volume receiving high doses to the maxilla in the range V55_LEM_–V60_LEM_, and the dose bath at lower doses. In this regard, CIRT in general for its physical and biological peculiarities, and the spot scanning beam delivery technique, might represent an advantage as postulated in Ikawa et al. when treating radioresistant tumors that requires high doses (25). In our series, even if the latency time for the ORN onset was similar as in Sasahara et al. (13), ORN incidence was less probable, not only because of the shorter follow up time in our series (50 vs 79 months), but possibly for the active spot scanning technique (vs passive beam) routinely employed at CNAO for treatments.

Not surprisingly, in our study with ACC in different locations in the maxillofacial area, ORN was mainly found after CIRT at the sinonasal site. In this regard, in Koto et al., over the 458 patients with sinonasal malignancies treated with different CIRT prescription doses but more frequently with 64 Gy (RBE) in 16 fractions, maxillary ORN was the most frequent late toxicity together with visual impairment, with G3 ORN reported in 18 patients (4%) and grade 2 in 36 (8%) (27).

In our series, the presence of superior dental elements within the PTV correlated with the onset and development of maxillary ORN at univariate analysis. In Bhattacharyya et al. the number of teeth irradiated with more than 50 Gy (RBE) was a significant independent risk factor for the development of oronasal fistula, which is also a late complication of CIRT. In order to reduce the risk, the authors suggested minimizing the number of teeth at a maximum of 2 within the volume irradiated with more than 50 Gy (RBE) (20). These data, together with data with conventional photon RT on the impact of dental infection on ORN incidence (28), confirm the benefit of pre-CIRT prophylactic dental avulsion and oral hygiene control before CIRT and during follow up adopted in CIRT facilities around the world, and at our center. Acute mucositis developed during the CIRT course (also significantly correlated with ORN in our series) together with the high radiation dose to the maxilla alveolar bone portion, might lead to superficial and then deep periodontal disease, gingival recession with increase susceptibility to infection with progressive bone injury leading to ORN (24). The elimination of odontostomatological risk factors (traumatic, inflammatory, and infectious events) and not, before the start of CIRT and during follow up, is of considerable importance for the outcome of the treatments and the quality of life of the patients. Time management of teeth extractions should also be strictly observed both before and after CIRT, and dental extraction protocol should be based on the individual plan isodose maps in case is not avoidable. As previously reported, teeth extraction or surgical procedure whenever not amenable should be performed not earlier than 1 year after CIRT to avoid ORN progression to soft tissue and skin, and more serious sequelae with related deterioration of quality of life (29). According to the median time of onset of maxillary ORN in our analysis, it would be advisable to wait at least for 24 months after CIRT before any bone intervention. Conservative measures are recommended for early stage ORN, reserving surgery at later stages when no other medical options are available.

In our series, we investigated also pre-CIRT surgery as risk factor for ORN. According to Marunick et al., pre-RT surgical risk involves tissue trauma, reduced blood supply, cell death, collagen lysis, and greater metabolic challenge to repair. Surprisingly, here postoperative CIRT was not correlated with increased ORN risk differently from Marunick et al., where only the 4 patients included in the study who had jointly received both definitive tumor resection and neutron RT developed ORN at the residual maxillary orbital complex (30).

In summary, among all the toxicities of patients treated with CIRT in the head and neck area, ORN is one of the most serious and debilitating chronic post-treatment sequelae. Here, we have pointed out the V55_LEM_–V60_LEM_ irradiated volumes as most important factor correlated with ORN. The limitations of our study include the small patient number and the short follow up. For the future, clinical trials with larger patients’ number and longer follow up are warranted. Based on our experience enriched by literature data, we might further summarize the recommendations for the optimal strategies to reduce ORN risk onset when irradiating tumors at the skull base, especially in facilities using the LEM-based CIRT: 1) to perform dental examination and prophylactic extraction procedures before CIRT if necessary, and close dentist follow up for all the 2-year period after CIRT more at risk of developing ORN, especially in case of patients with the maxilla covered for large volume by dose higher than 55 Gy (RBE); 2) to maximize the dose conformity to the maxilla by reducing volumes irradiated with dose higher than 55 Gy (RBE) and the dose bath below 50 Gy (RBE); 3) to avoid teeth in the PTV especially if the tumor does not involve the maxilla, and in case it is inevitable, to include not more than 2 teeth in the high dose PTV; 4) to prefer conservative measures and avoid surgical traumatic procedures after ORN onset. In case of unamenable necrosectomy after CIRT, our recommendation might be to remove the bone around the necrotic tissue by including at least the V55 _LEM_ isodose and higher, in order to facilitate tissue post-surgical healing.

## Data Availability Statement

The raw data supporting the conclusions of this article will be made available by the authors, without undue reservation.

## Ethics Statement

The studies involving human participants were reviewed and approved by the Ethical committee San Matteo Hospital, Pavia (Italy). The patients/participants provided their written informed consent to participate in this study.

## Author Contributions

Conceptualization: BV, SR, and EO. Methodology and statistics: BV, SR, SM, EO, MM, and SR. Writing: MM and BV. Review and Editing: BV, SR, MM, MB, SRO, RI, AMC, SI, LP, MC, SM, and EO. Supervision: BV and EO. All authors listed have made a substantial, direct, and intellectual contribution to the work and approved it for publication.

## Conflict of Interest

The authors declare that the research was conducted in the absence of any commercial or financial relationships that could be construed as a potential conflict of interest.

## Conflict of Interest

The authors declare that the research was conducted in the absence of any commercial or financial relationships that could be construed as a potential conflict of interest.

## Publisher’s Note

All claims expressed in this article are solely those of the authors and do not necessarily represent those of their affiliated organizations, or those of the publisher, the editors and the reviewers. Any product that may be evaluated in this article, or claim that may be made by its manufacturer, is not guaranteed or endorsed by the publisher.
